# Clinical experience with Long-Acting Injectable Antipsychotics (LAIs) in child and adolescent patients

**DOI:** 10.1192/j.eurpsy.2026.11140

**Published:** 2026-07-31

**Authors:** A. Merino Iglesias, C. González Navajas, S. González Álvarez, C. Lezcano González, M. Graell Berna

**Affiliations:** Psychiatry, Hospital Infantil Universitario Niño Jesús, Madrid, Spain

## Abstract

**Introduction:**

Long-acting injectable antipsychotics (LAIs) have undergone significant development in recent years, becoming useful tools to facilitate threpautic adherence. While some studies have demonstrated their effectiveness in the early stages of psychosis, most studies focus on adult populations, with few studies in children and adolescents.

**Objectives:**

In this study we will analyze our clinical experience with LAIs in children and adolescents.

**Methods:**

Longitudinal retrospective and descriptive study of patients admitted to a short-stay inpatient unit for children and adolescents in the Community of Madrid, between October 2024 and October 2025, who required treatment with LAIs.

A sample of 13 patients was selected, collecting both sociodemographic variables (age, gender) and clinical variables (substance use, associated diagnoses, antipsychotic used and dose, adherence and adverse events, number of readmissions and emergency department visits, mean time elapsed since administration, and use as monotherapy). A descriptive analysis of the data was performed.

**Results:**

The LAIs were applied to a total of 13 patients: 15% (n=2) were women, and 85% (n=11) were men. The mean age of the patients was 15.2 years. Regarding diagnosis: 23% (n=3) presented with “psychotic disorders”, 31% (n=4) with “autism spectrum disorders”, and 31% (n=4) with associated intellectual disability.

Behavioral problems were present in 77% of cases (n=10). 54% (n=7) presented with active substance use, of whom: all (100%) used cannabis, and more than half (57%) also used other substances.

The most frequently used antipsychotics were:

- Monthly aripiprazole (62%, n=13), with doses of 400 mg (46%, n=6) and 300 mg (15%, n=2).
- Monthly paliperidone (23%, n=3), with a maintenance dose of 100 mg in all cases.
- Monthly risperidone (15.4%, n=2) with doses of 75 mg (7.7%, n=1) and 100 mg (7.7%, n=1).

During the study period, hospital admissions for psychiatric decompensation occurred in 23% of cases (n=3), and emergency room visits in 46% of cases (n=6).

Regarding monotherapy: in 92% of patients (n=12), the injectable antipsychotic was used as monotherapy, while in 8% of cases (n=1), more than one antipsychotic was required.

No severe adverse effects occurred in any of the patients, and 100% of the patients analyzed maintained adherence throughout the follow-up period. The mean time elapsed since the start of administration was 6.9 months.

**Conclusions:**

In the analyzed sample, LAIs showed good tolerability and adherence data. Furthermore, they proved effective in preventing decompensation and hospital admissions in various clinical profiles.

Although further studies are needed to evaluate their systematic use in children and adolescents, they could represent a useful alternative in patients with poor treatment adherence, in the same way as in adults.

**Disclosure of Interest:**

None Declared

